# Held out wings RNA binding activity in the cytoplasm during early spermatogenesis

**DOI:** 10.1038/s42003-025-09435-4

**Published:** 2026-01-12

**Authors:** Michaela Agapiou, Karl Norris, Tayah Hopes, Eilidh Ward, Fruzsina Hobor, Amanda Bretman, Thomas A. Edwards, Julie L. Aspden

**Affiliations:** 1 School of Molecular and Cellular Biology, Faculty of Biological Sciences, University of Leeds, Leeds, UK; 2 LeedsOmics, University of Leeds, Leeds, UK; 3 Astbury Centre for Structural Molecular Biology, University of Leeds, Leeds, UK; 4 School of Biology, Faculty of Biological Sciences, University of Leeds, Leeds, UK; 5 College of Biomedical Sciences, Larkin University, Miami, FL USA

**Keywords:** RNA, Stem-cell niche

## Abstract

Held out wings (HOW) is an RNA-binding protein essential for spermatogenesis in *Drosophila melanogaster*. HOW is a signal transduction and activation of RNA (STAR) protein, regulating post-transcriptional gene expression. The characteristics of RNA-binding by the conserved short cytoplasmic isoform, HOW(S), are unknown. In vivo RIP-seq identified 121 novel transcripts bound by HOW(S) in germ stem cells and spermatogonia, many with signal transduction functions. (A/G/U)CUAAC motifs were enriched in 3’-UTRs and GCG(A/U)G in 5’-UTRs. HOW binds with high affinity to sites containing CUAAC motifs from *lola* and *hipk* mRNAs. The binding of cytoplasmic HOW(S) specifically to CUAAC motifs in 3’-UTRs can result in stabilization or destabilization of mRNAs and translational regulation potentially depending on position of binding, number of sites and interacting partners. This study provides new insight into STAR protein-RNA interactions and functions in spermatogenesis.

## Abstract (web summary)

Identification and characterization of cytoplasmic STAR protein-RNA interactions in D. melanogaster testis germ stem cells and functional impact of binding, providing new insights into the importance of RNA-protein networks during spermatogenesis.

## Introduction

Post-transcriptional gene regulation plays an essential role in stem cells, contributing to pluripotency, differentiation and self-renewal. Molecular signals contribute to germ stem cell regulation through a variety of RNA processes controlled by a network of RNA-binding proteins, including the signal transduction and activation of RNA (STAR) protein family. STAR proteins characteristically contain a single RNA-binding STAR domain, composed of one maxi-K-homology (KH) domain, and one or two flanking regions^1^. As a result of their functions in signal transduction pathways, the family contributes to the regulation of gene expression during key stages of development. For example, the STAR protein Quaking (QKI) regulates RNA processing during gametogenesis and myelination in mammals^2^. RNA-binding proteins in general are important throughout spermatogenesis, including for stem cell maintenance and meiosis, across a variety of organisms^3^.

Held out wings (HOW) is the ortholog of QKI in *Drosophila melanogaster*. HOW is essential, but partial loss of function mutants exhibit a variety of phenotypes including defective wing development, glial maturation, tendon maturation and male sterility^4–7^. HOW regulates RNA processing, including pre-mRNA splicing, localisation and mRNA degradation^8–10^. There are multiple protein isoforms of HOW, produced through alternative splicing, all of which contain the STAR domain but differing at the C-terminus^11^(Supplementary Fig. 1A). The different isoforms of QKI and HOW are differentially localised within cells, and the shortest isoforms, QKI-6 and HOW(S), are predominantly cytoplasmic (Supplementary Fig. 1B), where their function is unknown. The HOW and QKI ortholog in *C. elegans*, GLD-1, has a single cytoplasmic isoform that regulates mRNA translation in germ cells^12^. Mirroring QKI’s expression in mammals, HOW is expressed in *D. melanogaster* testis, and it is important during spermatogenesis^7^. In germ cells, HOW’s expression is unusual and highly restricted to the earliest stages of gametogenesis, germline stem cells (GSCs), child cells (gonialblasts), and two-cell spermatogonial cysts^7^. HOW contributes to the maintenance of GSCs; without HOW, GSCs in the testis do not survive^7,13^. Over-expression of either HOW(L) or HOW(S) is insufficient to fully rescue the loss of GSC phenotype in how^r17/r4^ mutants^7^. This suggests that HOW(L) and HOW(S) perform different functions, likely the result of their differing sub-cellular localisation, nuclear and cytoplasmic, respectively. Expression of HA-tagged HOW(S) in the testis indicates that this isoform is restricted to the cytoplasm in germ cells^7^, which is consistent with HOW(S) lacking the nuclear localisation signal at the C-terminus that targets HOW(L) to the nucleus. The restricted expression of HOW during spermatogenesis suggests a specific function in regulating the balance between stem cell maintenance versus the proliferation and differentiation of spermatogonia.

The majority of work on HOW thus far has focused on HOW(L) and its role in nuclear RNA processing events. Current understanding of HOW-RNA binding comes from a handful of mRNAs (e.g. *dpp, miple1, bam*), which contain 3’-UTR ACUAA motifs typically bound by HOW(L)^14^. The only mRNA previously known to be bound specifically by HOW(S) rather than HOW(L) is *dgrasp* in oocytes^15^. An optimal binding preference has yet to be characterised for HOW, but a consensus of NCUAACN has been generated from in vitro binding experiments^16^. Here, we sought to identify novel mRNA targets of HOW in the testis, specifically by HOW(S) in the cytoplasm, to understand the binding characteristic of this interaction in vivo.

## Results

### Expression and pull-down of cytoplasmic HA-tagged HOW(S) in testis germ cells

**Fig. 1:**
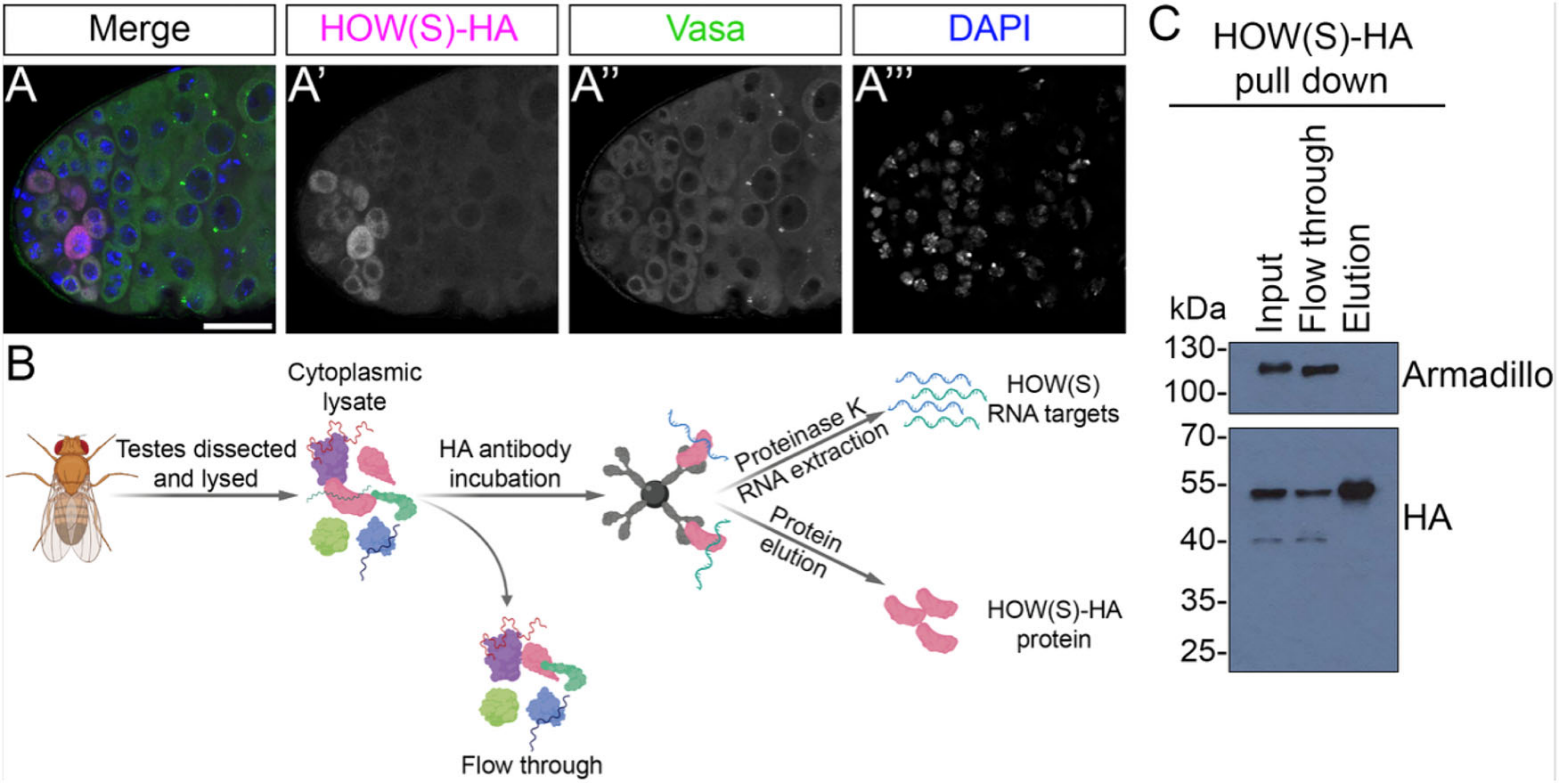
Expression and pull-down of HA-tagged HOW(S) from the cytoplasm of germ cells. **A** Confocal images showing cytoplasmic expression of HOW(S)-HA (magenta) in GSCs and spermatogonia, driven by the nanos-GAL4 line. Germ cells are positive for vasa (green), and blue is DAPI. **B** Schematic of HOW(S)-HA RIP-seq and generation of samples (generated with Biorender). **C** Western blot showing specific depletion of HOW(S)-HA from cytoplasmic testis lysate ‘input’ and enrichment in the elution using anti-HA beads for 1 biological replicate. Armadillo is used as a nonspecific control.

To understand the role of HOW(S) and the RNAs it binds in the cytoplasm, HA-tagged HOW(S) was expressed in the early stages of spermatogenesis in *Drosophila melanogaster* testes. This was achieved by using the UAS/GAL4 system, whereby HOW(S)-HA expression was driven by *nanos*-GAL4, therefore expressing HOW(S)-HA in GSCs and early spermatogonia (Fig. 1A). This HA-tagged HOW(S) is localised to the cytoplasm of these cells (Fig. 1A), recapitulating the presence of cytoplasmic HOW in these specific germ cells^7,13^. We were able to successfully pull down HOW(S)-HA from the lysates of these testes using anti-HA beads (Fig. 1B). To isolate RNA bound to HOW(S), RNP-IPs were performed from large-scale testes lysates in triplicate (Fig. 1C and Supplementary Fig. 2A–C). RNA from the HOW(S)-HA pull-down was purified along with RNA from input testis lysates and RNA from pull-downs performed from the *nanos*-GAL4 parent testes, which had no HA-tagged HOW(S) (Supplementary Fig. 2D). These RNA samples then underwent RNA-seq.

### HOW(S) bound RNAs are enriched for signalling functions

**Fig. 2:**
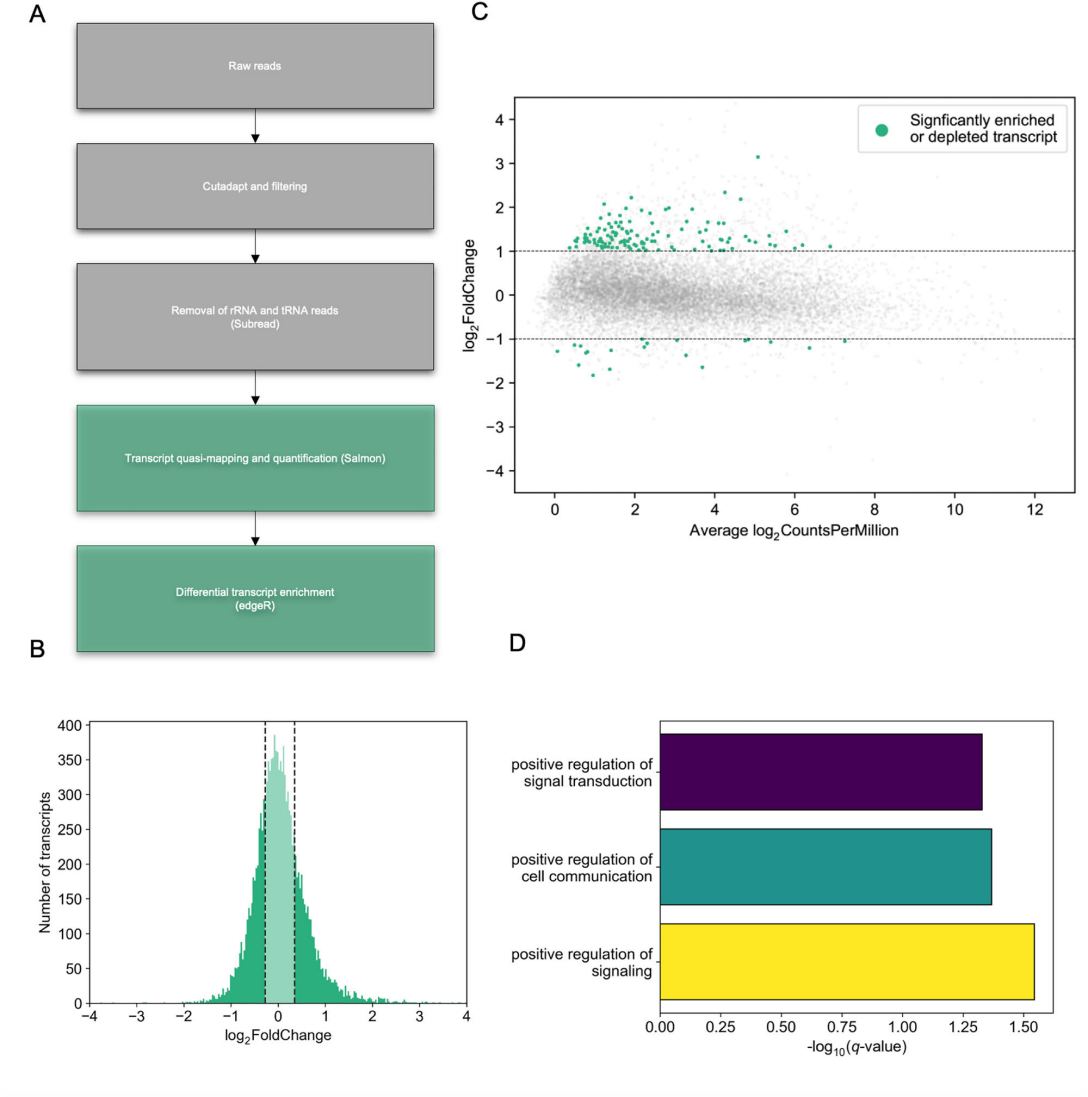
Enrichment of HOW(S) bound RNA, with a role in signal transduction. **A** Pipeline to identify RNAs specifically bound to HOW(S). **B** Histogram from differential enrichment analysis of pull-down versus input, middle quartiles are shaded in lighter green. **C** Scatter plot from differential transcript analysis. Significantly enriched or depleted transcripts are those with log_2_(fold change) of ≥1 or ≤−1 (dotted lines), and an FDR-corrected *p* value ≤0.05, additionally nonspecific background RNAs enriched in the parental nanos-GAL4 RIP-seq sample were subtracted, using three independent biological replicates of pull-downs. Specifically enriched or depleted RNAs from the HOW(S) RIP-seq are highlighted in green. **D** GO term analysis reveals over-representation of signalling-related terms in transcripts bound by HOW(S).

To identify which RNAs were specifically bound to HOW(S)-HA, differential transcript enrichment analysis was performed on RNAs in HOW(S)-HA pull-down compared to those present in input lysate (Fig. 2A, Supplementary Data 1, and Supplementary Fig. 3A, B). The majority of transcripts showed no enrichment, having a log_2_(Fold Change) between −0.5 and 0.5 (Fig. 2B). We defined enriched transcripts as those with a log_2_(fold change) ≥1 and an FDR-corrected *p* value ≤0.05. Through this analysis we identified 403 transcripts enriched in the HOW(S)-HA pull-down RNA compared with input RNA. Non-specific background RNAs enriched in the parental *nanos*-GAL4 RIP-seq sample (with no HOW(S)-HA present) were then subtracted, leaving those RNA transcripts specifically bound to HOW(S)-HA. 121 mRNAs were identified as specifically enriched by HOW(S) RIP-seq (Fig. 2C—green dots and Supplementary Data 1). To understand the role of the RNAs bound by HOW(S), GO analysis was performed on the genes corresponding to these HOW(S) bound mRNAs. This revealed an enrichment for genes with functions in cell signalling, specifically in cell communication and signal transduction e.g. *hipk* (homeodomain interacting protein kinase) and *CycG* (cyclin G) (Fig. 2D and Supplementary Fig. 3C). This is entirely consistent with HOW’s membership of the STAR family of proteins.

### CUAAC motif enriched in 3’-UTRs of HOW(S) bound RNAs

**Fig. 3:**
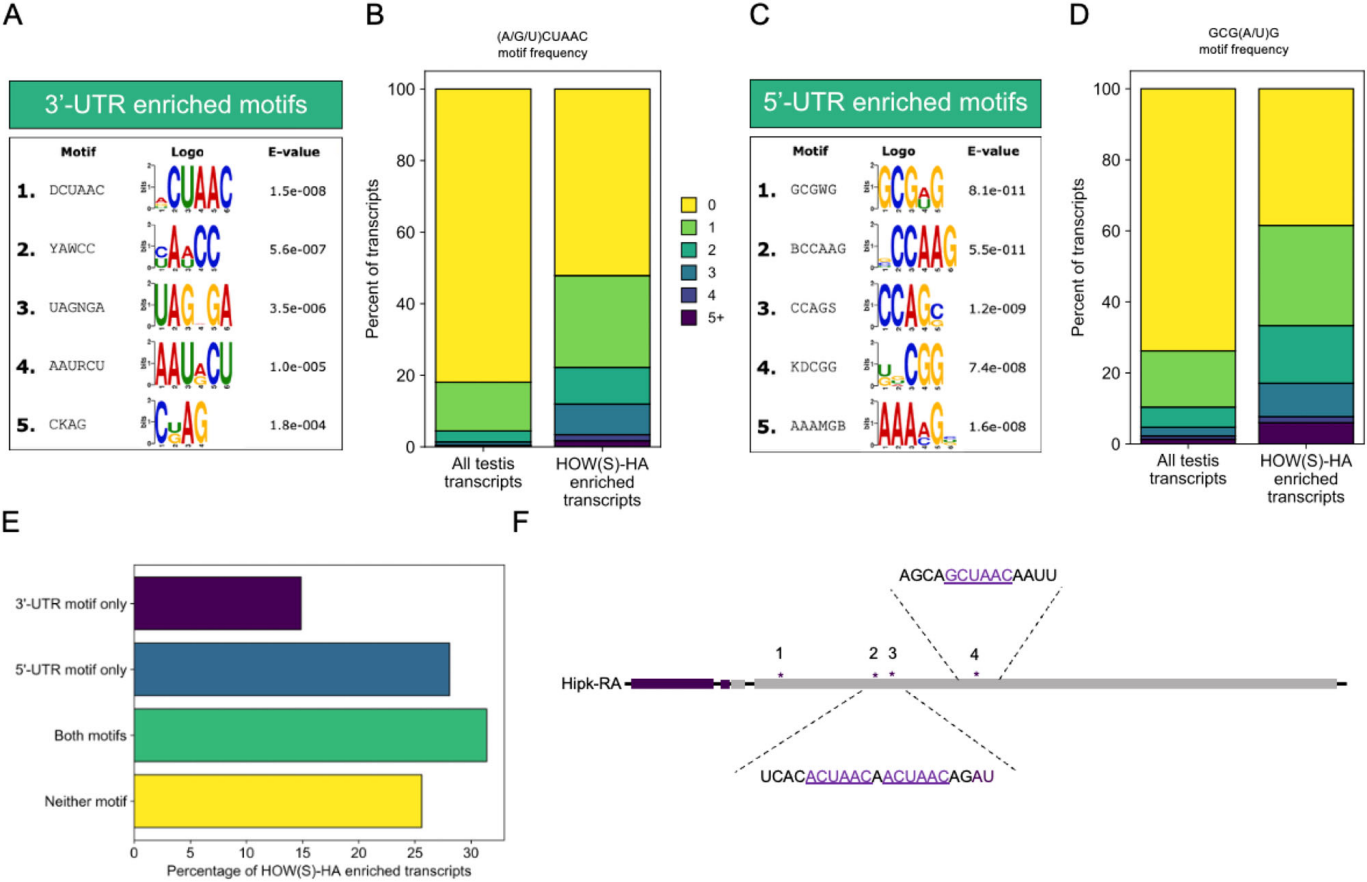
Enrichment of CUAAC motifs in HOW(S) bound RNAs. **A** Motifs found to be enriched in 3’-UTRs of mRNAs bound by HOW(S). **B** Frequency at which (A/G/U)CUAAC motif identified in 3’-UTR of HOW(S) bound transcripts compared to the 3’-UTRs of all testis expressed transcripts. **C** Motifs found to be enriched in 5’-UTRs of mRNAs bound by HOW(S). **D** Frequency at which GCG(A/U)G motif identified in 5’-UTR of HOW(S) bound transcripts compared to the 5’-UTRs of all testis expressed transcripts. **E** Percentage of transcripts bound by HOW(S) that had either of the top UTR motifs, both, or neither in their respective UTRs. **F** Schematic of the 3’ end of hipk transcript, purple is CDS, grey is UTR, and asterisks mark (A/G/U)CUAAC sites in the 3’-UTR.

To determine the nature of HOW(S) binding to the 121 transcripts identified by RIP-seq, motif analysis was performed on these mRNA targets. Within the 3’-UTRs, the most enriched motif was found to be (A/G/U)CUAAC (Fig. 3A), with 46% of the HOW(S)-HA bound mRNAs containing this motif (Fig. 3B). The second most enriched motif in the 3’-UTRs was YAWCC or (C/U)A(A/U)CC, and found in 79% of bound mRNAs (Supplementary Fig. 3D). (A/G/U)CUAAC is very similar to the other previously identified HOW binding sequence in *stripe* and *dpp* mRNAs: ACUAA^14^. Rather than characterising a small number of HOW targets and identifying individual binding sites, the (A/G/U)CUAAC motif generated here is from a much larger number of RNAs bound by HOW(S) in the cytoplasm.

Other distinct motifs were found enriched in the 5’-UTRs of HOW(S) bound mRNAs, including GCG(A/U)G (Fig. 3C), but these do not show similarity with previously identified HOW binding sites^9,14,16^. Although these are unlikely to be HOW(S) binding sites, because their sequence differs dramatically from the previously characterised ACUAA motif, these motifs may be important for HOW(S) RNA-protein complexes as they are highly enriched in the HOW(S) bound mRNAs—the GCG(A/U)G motif is present in 60% of HOW(S) bound mRNAs (Fig. 3D). Instead, these motifs may represent sequences that are bound to another RNA-binding protein (RBP) associated with HOW(S), since RIP-seq will identify both indirect and direct interactions. This type of co-binding has been demonstrated for HOW(L), which can interact with the RBP SXL, with the two proteins binding distinct sequences in the 5’-UTR of *msl-2*^9^. We also observed that 31% of HOW(S) bound mRNAs contain both the top 5’- and top 3’-UTR enriched motifs in the respective UTRs (Fig. 3E), which indicates that HOW(S) molecules could bind to both UTRs of an mRNA transcript simultaneously, potentially regulating the mRNA in numerous ways. This has been shown for other RBPs during development e.g. SXL during sex determination^17^.

Further analysis of the HOW(S) bound transcripts that have the (A/G/U)CUAAC motif reveals that 21% of them contain multiple repeats of this motif in their 3’-UTRs, which is substantially higher than that found in all testis expressed transcripts (Fig. 3B). RBPs often bind RNAs in a modular manner, and HOW, along with many of the STAR proteins, dimerise^18^. Thus, multiple binding sites in HOW(S) bound transcripts could indicate a modular binding mode. This pattern is also seen for the top 5’-UTR enriched motif GCG(A/U)G (Fig. 3D), indicating that this too may represent a modular RBP binding region.

One such mRNA which has multiple predicted binding sites is *hipk*. Hipk protein functions in multiple signalling pathways, including Notch^19^, Hippo^20,21^ and the JAK-STAT pathway^22^, and is essential for germline development in *C. elegans* and mouse spermatogenesis^23,24^. *Hipk-RA* transcript (FBtr0072552) was identified as enriched in the HOW(S)-HA RIP-seq and contains four (A/G)CUAAC sites within its 3’-UTR (Fig. 3F), therefore likely represents a key HOW(S) target with downstream roles in signalling pathways essential in maintaining the stem cell niche and cell fate.

### Recombinant HOW-STAR domain binds enriched motifs within HOW(S) bound RNAs with high affinity

**Fig. 4:**
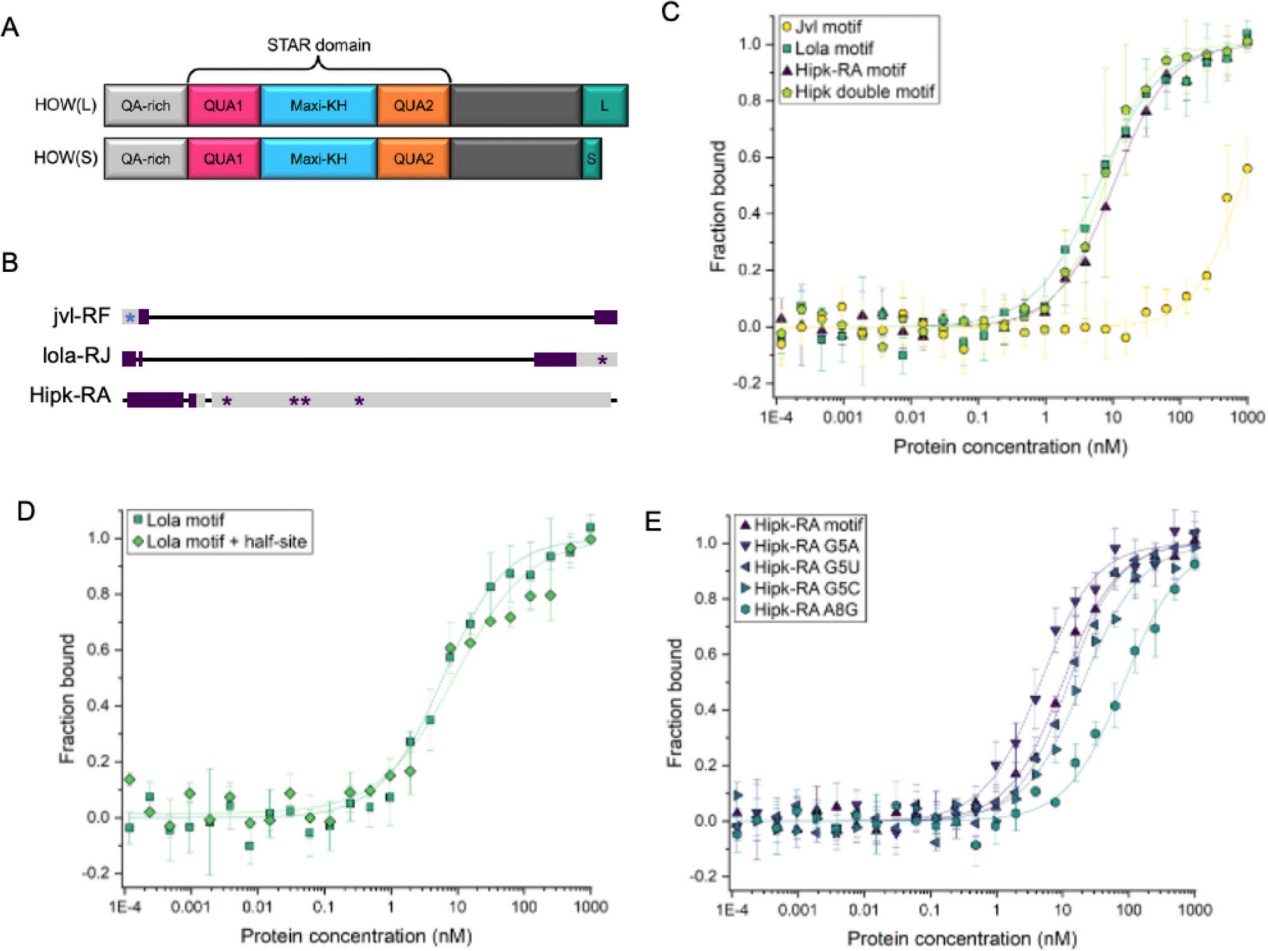
HOW KH domain binds to HOW(S) targets with high affinity and specificity. **A** Schematic of two HOW protein isoforms, indicating the STAR domain, which was expressed recombinantly (codon optimised STAR domain, amino acids 72–266 (HOW-PB; FBpp0083576). **B** Schematic of three transcripts bound by HOW(S): the 5’ end of jvl-RF, and 3’ ends of lola-RF and Hipk-RA. Asterisks mark the 5’-UTR (blue) and 3’-UTR (purple) motifs. Purple bars are CDS, grey is UTRs. **C** FA binding plots from jvl motif, lola motif and Hipk-RA motif, as well as the Hipk double site. The higher the protein concentration required to achieve complete RNA binding indicates weaker binding. Error bars represent standard deviation between 3 experimental replicates carried out on same FA plate. **D** FA binding plots for lola motif and lola motif + half-site. **E** FA binding plots for mutations in the Hipk-RA motif. Error bars represent standard deviation between three experimental replicates (distinct binding reactions) carried out on same FA plate.

To test the binding of HOW to the motifs identified as enriched from RIP-seq we recombinantly expressed HOW’s STAR domain (Fig. 4A and Supplementary Fig. 4A–C) and performed fluorescence anisotropy (FA) binding assays. This domain is common between all protein isoforms of HOW including HOW(S) and HOW(L) (Fig. 4A)^25^. FA measures the kinetics of protein-RNA binding by detecting changes in the tumbling rate of fluorescently labelled RNAs by the degree of depolarisation^26^. Three different mRNAs that we identified as being bound by HOW(S) in GSCs and early spermatogonia were selected to test HOW binding to sequences from within these transcripts that were identified as highly similar to the enriched motifs (*jvl-RF, lola-RF and Hipk-RA)*.

A site was selected from the 5’-UTR of *jvl* transcript (FBtr0305694)(Fig. 4B) that contained the most highly enriched 5’-UTR motif within HOW(S) bound mRNAs: GCGUG (Table 1-Jvl). FA analysis of this Jvl RNA oligo revealed that HOW-STAR did not bind to this site in the low nanomolar range (Fig. 4C and Table 1). This suggests that although the GCG(A/U)G motif is highly enriched in the 5’-UTRs of HOW(S) bound mRNAs, it is unlikely to be an in vivo HOW(S) binding site. An alternative explanation could be that HOW(S) interacts with a second RBP, which provides the RNA-binding specificity for these interactions. One potential candidate is SLIRP1 protein, which has a binding motif that is highly similar to the one found in the 5’-UTR of HOW(S) bound RNAs: GCG(U/C)(G > A/C/U)^16^ and is highly expressed in the testis.

**Table 1:** Summary of HOW binding affinities for HOW(S) predicted binding sites

Oligo name	Sequence	Apparent *K*_D_ (nM) to 3 sig. fig.	Standard error (±)
Jvl	GUUGGCGUGUUUU	803	1064
Lola	ACACACUAACUCGU	6.05	0.62
Lola + half-site	ACACACUAACUCGUAACUAUG	9.25	1.57
Hipk-RA	AGCAGCUAACAAUU	10.1	0.73
Hipk-RA G5A	AGCA**A**CUAACAAUU	4.65	0.56
Hipk-RA G5U	AGCA**U**CUAACAAUU	12.4	1.25
Hipk-RA G5C	AGCA**C**CUAACAAUU	20.4	2.86
Hipk-RA A8G	AGCAGCU**G**ACAAUU	85.3	22.1
Hipk double motif	UACAACUAACAACUAACAGAU	7.46	1.76

Sequence of oligos based on the predicted binding sites within HOW(S) targets, along with apparent *K*_D_ and standard error calculated from FA. The higher the *K*_D_ value, the weaker the HOW-RNA binding. Sequences that match the top motifs from the motif enrichment analysis are underlined, and nucleotides mutated are in bold.

We also tested RNA oligos based on regions from *lola* and *hipk*, two transcripts bound by HOW(S), which contain sequences matching the 3’-UTR HOW(S) motif (Fig. 4B). The *Lola* element contains an ACUAAC motif (Table 1) and HOW-STAR exhibits low apparent *K*_D_ value (6.05 ± 0.62 nM), which reflects high affinity binding to this RNA oligo by FA (Fig. 4C and Supplementary Fig. 4D). Just downstream of this motif is a potential half-site and such half-sites have been suggested to enable stronger binding by STAR proteins^27^. However, the RNA oligo including this additional half-site did not increase the affinity of HOW-STAR for the HOW binding site, as there is little difference in apparent *K*_D_ between Lola (6.05 ± 0.62 nM) and Lola + half-site RNA oligos (9.25 ± 1.57 nM), (Fig. 4D and Table 1). Therefore, we focused simply on HOW(S) binding sites with a complete motif.

Multiple putative binding sites were found in *Hipk-RA* mRNA that match the 3’-UTR enriched motif (Fig. 4B), so we characterised the binding of HOW(S) to RNA oligos containing the sequences of two of these regions (Fig. 3F). The first site (Table 1: Hipk-RA) contains a GCUAAC motif (Table 1) and exhibits high affinity binding (10.1 ± 0.73 nM) with HOW-STAR (Fig. 4C). Our motif enrichment analysis indicated that the first position of the top 3’-UTR motif, (A/G/C)CUAAC, appeared to be the most flexible (Fig. 3A). Therefore, the G from this GCUAAC motif was mutated to A, C or U, and binding tested to understand the effect on HOW-STAR binding affinity in the context of the same 14mer oligo used in these FA experiments (Table 1: Hipk-RA G5A, G5U, G5C). Lower apparent K_D_ values reflect stronger HOW-RNA binding. This revealed that there is a preference for adenosine (4.65 ± 0.56 nM) at this site over guanosine (10.1 ± 0.73 nM), uridine (12.4 ± 1.25 nM) and cytidine (20.4 ± 2.86 nM) (Fig. 4E and Table 1). This is consistent with the motif enrichment analysis, where adenosine is more commonly found compared with other nucleotides (Fig. 3A). To show specificity of HOW binding to these NCUAAC motifs, the adenosine in the fourth position was mutated to a guanosine (Table 1: Hipk-RA A8G), which resulted in a large decrease (85.3 ± 22.1 nM vs ACUAAC in Hipk-RA 10.1 ± 0.73 nM) in HOW-STAR binding affinity (Fig. 4E and Table 1). This is also consistent with the mutational analysis performed on HOW’s binding site in *stripe*’s 3’-UTR, which can be bound by either HOW(S) or HOW(L)^14^.

The second Hipk-RA site contains two HOW(S) motifs in close proximity in the 3’-UTR (Table 1: Hipk double motif). The binding of HOW-STAR with this region, was of very similar affinity (7.46 ± 1.76 nM) to the site only containing one CUAAC motif (Hipk-RA 10.1 ± 0.73 nM), suggesting that only a monomer of HOW is binding to this region, rather than dimer (Fig. 4C and Table 1). Other STAR proteins can bind as dimers and this may be the case for HOW but at more distant sites than those within this ‘double motif’ (Table 1), as a distance of >15nt has been shown to be required for dimer binding of other STAR proteins (e.g. GLD-1, QKI and Sam68)^28^, although avidity effects may also be in play here. For example, a HOW dimer binding to one site might have a *K*_D_ of 10 nM, but a HOW dimer binding to two sites might have a *K*_D_ of 2 nM (so even better than double the strength of binding), because of increased local concentration of the second molecule of HOW.

### HOW(S) binding to mRNAs containing CUAAC motifs act to stabilise mRNAs

**Fig. 5:**
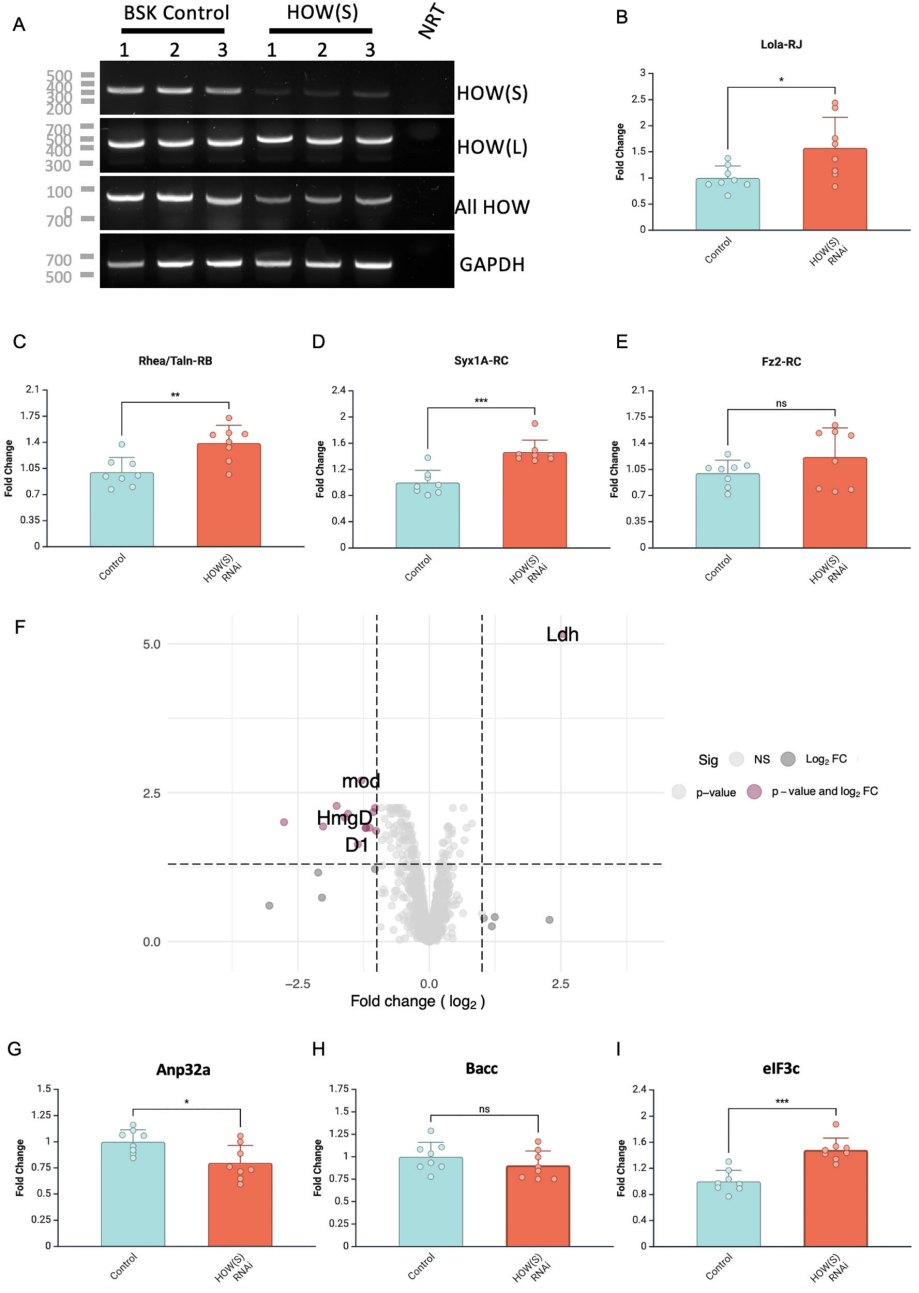
Functional impact of HOW(S) binding on mRNA transcript and protein levels in S2 cells. **A** Agarose gel of RT-PCR showing HOW(S) specific knockdown, compared to HOW(L) and all HOW isoforms, and GAPDH control, with DNA ladder in bp on the left for three replicates (1–3) along with no RT control (NRT). RT-qPCR of **B** Lola-RJ, **C** Rhea/Tain-RB, **D** Syx1A-RC, **E** Fz2-RC, mRNA levels in control compared to HOW(S) RNAi. **F** Volcano plot of proteomic differences between control and HOW(S) RNAi with log_2_ fold change ≥1 and ≤−1 cut-off and *p*_adj_ <0.05 cut-off. Points labelled are those proteins whose mRNAs were identified as bound by HOW(S) RIP-Seq in testis germ stem cells. RT-qPCR of **G** Anp32a, **H**) Bacc and **I** eIF3c mRNA levels in control compared to HOW(S) RNAi. RT-qPCR error bars represent standard deviations of *n* = 8 biological replicates *p value <0.03, **p value <0.005 and ***p value <0.001 unpaired *t*-test.

To dissect the functional consequence of HOW(S) binding we performed HOW(S) RNAi knockdown in *Drosophila* S2 cells. RNAi duplex treatment resulted in specific knockdown of HOW(S) and not HOW(L) isoform (Fig. 5A). RT-qPCR was performed on four transcripts shown to be bound by HOW(S) in the testis germ stem cells. Three of these transcripts *Lola-RJ*, *Syx1A* and *Talin/Rhea-RB* exhibited significantly increased mRNA abundance upon HOW(S) RNAi knockdown (Fig. 5B–D). This includes *lola* mRNA, which contains HOW motif ACUAAC in its 3’-UTR, shown to be a high-affinity HOW-STAR domain binding site by FA. Fz2 mRNA levels were not significantly affected by HOW(S) RNAi (Fig. 5E). Together, this indicates that HOW(S) binding can result in the destabilisation of mRNAs.

Given that the transcriptome of S2 cells is substantially different from testis germ stem cells, we also performed an unbiased analysis of proteomic changes upon HOW(S) RNAi knockdown. This revealed 1 protein upregulated and 14 proteins downregulated, with cut-offs of log_2_ fold change ≥1 and ≤−1 and *p*_adj_ < 0.05 (Fig. 5F and Supplementary Data 2). The mRNAs encoding these proteins were analysed to determine if they contained motifs found to be enriched in germ stem cell RIP-Seq. On inspecting all possible transcripts generated from these 15 regulated genes, ten were found to contain either the top 5’-UTR or 3’-UTR motifs, with seven containing CUAAC within the 3’-UTRs or 5’-UTRs (Supplementary Data 2). The levels of the three transcripts, which contained 3’-UTR CUAAC motifs and were significantly downregulated at the protein level (Fig. 5F and Supplementary Fig. 5A–C) were analysed by RT-qPCR (Fig. 5G–I). *Mapmodulin* (Anp32a) RNA levels were significantly reduced upon HOW(S) RNAi (Fig. 5G), *Bacc* mRNA levels were unaffected (Fig. 5H), whilst *eIF3c* mRNA levels were significantly increased (Fig. 5I). Surprisingly, those mRNAs identified by testis RIP-Seq and shown to have altered mRNA levels upon HOW(S) RNAi, did not significantly change at the protein level (Supplementary Fig. 5D–F). Together, this data suggests that cytoplasmic HOW(S) binding to 3’-UTR CUAAC motifs can act to stabilise or destabilise mRNA levels depending on the transcript. HOW(S) also has the potential to translational regulate mRNAs since *Bacc* mRNAs were unaffected, whilst protein levels were. Overall, these data indicate that cytoplasmic HOW(S) binding has varied effects on post-transcriptional gene regulation, potentially depending on the position of binding, number of sites and interacting partners.

## Discussion

Overall, we have identified 121 novel targets of cytoplasmic HOW(S) in testis germ cells, several of which are involved in signal transduction. Within these HOW(S) bound RNAs, the (A/G/U)CUAAC motif is enriched in 3’-UTRs and GCG(A/U)G in 5’-UTRs. This 3’-UTR motif is similar to potential HOW binding sites identified in *stripe* and *dpp* mRNAs, as well as the consensus sequences of other STAR proteins. The STAR domain of HOW has strong (nanomolar *K*_D_) affinity for RNA elements containing the CUAAC motif from *lola* and *hipk* mRNAs, but not with the GCG(A/U)G 5’-UTR motif. The motifs tested for HOW-STAR domain binding here are similar to those previously characterised (ACUAA) to be bound by HOW(L) in *dpp, miple1, bam* mRNAs^14^. Due to previous challenges in generating soluble full-length recombinant STAR proteins, HOW’s STAR domain alone was used in FA experiments to analyse RNA binding. Therefore, a limitation of our work could be that binding affinities for target mRNAs might be different if the additional domains were present. Specifically, STAR proteins are known to be regulated by signalling pathways via post-translational modifications, which can influence RNA-binding affinity (e.g. Sam68)^29^. These binding sites could potentially be bound by HOW(L) in the nucleus; however, our RIP-Seq has shown them to be bound by HOW(S) in the cytoplasm. The functional consequence of these different HOW-RNA interactions in these two compartments are likely to be different, given the varied roles HOW plays in RNA biology. RNAi S2 cells revealed that HOW(S) can contribute to mRNA stabilisation/destabilisation and translational control, whereas HOW(L) binding to transcripts in the nucleus can regulate splicing^5^, destabilise mRNAs^30^ or increase nuclear transcript retention^9^. Our work is consistent with the impact of HOW binding on *stripe* mRNA molecules in tendon cells. HOW(L) has a destabilising effect, which is dependent on its nuclear localisation, while cytoplastic HOW(S) stabilises^10^. Early in tendon cell differentiation, HOW(L) negatively regulates Stripe protein by retaining *stripe* mRNA in the nucleus, while the expression of HOW(S) later in embryogenesis promotes the release and stabilisation of *stripe* mRNA in the cytoplasm^10^. Potentially, a similar mechanism exists during spermatogenesis.

Together, these results provide new insight into STAR protein-RNA interactions and their potential importance to spermatogenesis. Given the importance of HOW to germ stem cells, understanding these novel cytoplasmic protein-RNA networks and their functional consequences will shed new light on how the balance of self-renewal and differentiation is regulated in stem cells.

## Methods

### Fly husbandry and stocks

Flies were kept in a 25 °C humidified room with a 12:12 h light:dark cycle and raised on 10 ml standard sugar-yeast-agar medium^31^. Crosses were generated by collecting unmated flies, then five to ten flies of each sex were placed into a vial together with grains of active baker’s yeast. UAS-HOW-S-HA line generously gifted by Prof T. Volk, *nanos*-GAL4 line used is #64277 from BDSC.

### Immunofluorescence

About 0- to 3- day-old unmated male flies were anaesthetised and dissected in 1X PBS. The following fixing, washing and staining steps were carried out with samples rotating. Testes were fixed with 4% paraformaldehyde in PBS for 40 min at room temperature (RT). Washed with 1X PBX (Supplementary Table 1) three times, blocked with blocking buffer (Supplementary Table 1) for 1 h at RT. Primary antibodies were used in 1X PBX and applied for 2 h at RT or overnight at 4 °C. Testes were washed three times and then incubated with secondary antibodies for 2 h at RT or overnight at 4 °C. This process was repeated for the next primary and secondary antibody pair. Samples were mounted with Vectashield antifade mounting medium with DAPI (Vector Laboratories). All antibodies used are described in Supplementary Table 2. Slides were imaged using a Zeiss LSM880 Upright Confocal Microscope with the 40X oil-immersion objective and Zen imaging software. The lasers used were Argon 488, DPSS 561 nm, and Diode 405 nm.

### Ribonucleoprotein immunoprecipitation

The reproductive systems of 0–3-day-old male flies (mated and unmated) were dissected in 1X PBS with 30 U/mL RNasin Plus RNase inhibitor (Promega). After ten pairs of testes were dissected, the tissue was snap frozen in liquid nitrogen. At least 1000 pairs of testes were collected per sample. Tissue was homogenised with an equal volume of RIP lysis buffer (Supplementary Table 1) with a micropestle and then with 23 23-gauge needle. Lysates were incubated on ice for 30 min, inverted halfway, and then snap frozen. Samples were thawed and centrifuged at 15,000×*g* for 15 min at 4 °C. About 20 µL or 10% of supernatants (whichever was less) was set aside and snap frozen as the ‘input’ samples.

RIP pull-downs were adapted from ref. ^32^. About 180 µL of anti-HA beads (Pierce, #88837) per sample were used and washed five times in ice-cold NT2 buffer (Supplementary Table 1) prior to usage. Beads were resuspended in NT2 buffer at 5.5 times the volume of the sample, and 0.2 U/μL of RNase inhibitor and EDTA, pH 8, to a final concentration of 20 mM were added. Beads and lysates were combined and incubated while tumbling for 1 h at RT. After incubation, the supernatant was taken as the ‘flow through’ samples and snap frozen. Beads were then washed five times in 1 mL NT2 buffer.

A quarter of the beads were eluted for protein samples; these were boiled at 95 °C for 10 min in 25 µL of 2X protein sample buffer (Supplementary Table 1). These samples were analysed by western blotting. The antibodies used for Western blots are described in Supplementary Table 3.

The remaining three quarters of the beads went through RNA elution and extraction. First, the elution samples were treated with 20 mg/mL proteinase K at 65 °C for 35 min. Then supernatants from this step, alongside proteinase K-treated ‘input’ samples, were treated with three times their volume in Trizol for 5 min at RT. About 0.15% (v/v) chloroform was added, and tubes were vigorously shaken for 15 s, incubated for 3 min at RT before being centrifuged at 12,000×*g* for 15 min at 4 °C. The aqueous phase was collected, and 1 volume of isopropanol was added along with 1 µL glycoblue and 0.3 M NaCl. The RNA was precipitated at −80 °C for at least 3 h. After precipitation, samples were centrifuged at 13,300×*g* for 20 min at 4 °C. The pellet was washed twice with 70% ethanol with 5 min centrifuge spin in between. After the final spin the supernatant was removed and the pellet was left to air-dry for 10–15 min. The pellet was resuspended in 18 µL nuclease-free water.

### Sequencing and computational analysis

#### Sequencing

The RNA from the lysates and elutions of three HOW(S)-HA samples and the *nanos*-GAL4 parental control was prepared using the Ribo-Zero rRNA Removal Kit (Illumina), followed by the TruSeq Stranded Total RNA Library Prep (Illumina). About 100 ng of each sample was pooled and sequenced on the same lane on the NextSeq 500 Illumina sequencer using the High Output Kit v2.5 (75 Cycles), i.e. 75 bp single-end sequencing.

#### Filtering

The adaptor sequence (AGATCGGAAGAGCACACGTCTGAACTCCAGTCAC) was trimmed from reads with Cutadapt (version 1.1)^33^. Reads were filtered out if the quality score was below 20 for 10% or more of the read. Filtering was done using the ‘Filter by quality’ tool in Galaxy (version 1.0.2)^34^. Next, rRNA and tRNA reads were removed using Subread (version 2.0.0)^35^. The tRNA fasta file was from release 6.22 of the *D. melanogaster* genome. The rRNA fasta file was from RiboGalaxy’s Shared Data library (UUID: 0f0983aa-3afb-4b5f-a417-23593a3df1ef).

### Transcriptome mapping

Transcriptome indexing and quasi-mapping was carried out using Salmon (version 0.14.2)^36^. A decoy-aware transcriptome was indexed with a pre-computed decoy sequence file provided by the Salmon developers. The transcriptome and decoy files used were based on the *D. melanogaster* genome from Ensembl release 97, which corresponds to FlyBase’s release 6.22. The k-mer size selected for indexing the transcriptome was 31. The reads remaining after filtering were quasi-mapped to the transcriptome with library parameter (-l) set to SR to correspond with the reverse-stranded library type. The k-mer parameter (-k) was set to 31.

#### Differential transcript enrichment

The following analysis was executed in RStudio, version 0.99.486, with R version 3.6.2^37^. Transcript counts from Salmon were imported using the tximport package (version 1.14.0)^38^ using txOut=TRUE and countsFromAbundance = 'scaledTPM' while importing. Differential enrichment was carried out with the edgeR package (version 3.28.0)^39,40^. Low-expression transcripts were filtered using the filterByExprfunction, libraries were normalised using calcNormFactors.

Two factors were defined for the HOW(S)-HA samples: condition and pairing. The condition referred to whether a sample was a total RNA or a pull-down RNA sample. Pairing refers to the three pairs of total and pull-down RNA samples. Thus, the design matrix was submitted as follows: model.matrix(~sample$condition + sample$pair). One factor was defined for the *nanos*-GAL4 samples to differentiate between the total RNA and pull-down RNA. The design matrix was submitted as: model.matrix(~group). For the HOW(S)-HA samples, dispersion was estimated with the design matrix taken into account. Then, testing for differential genes and transcripts was carried out with the quasi-likelihood *F*-test, as per the edgeR manual instructions. For the *nanos*-GAL4 samples, the exact test was used with the dispersion set as the square of the biological coefficient of variation (BCV). The BCV was set as the square root of the common dispersion from the HOW(S) data.

Finally, transcripts from the HOW(S)-HA RIP-seq data were classed as significantly enriched or depleted if they had an adjusted *p* value <0.05, had a log_2_(fold change) above 1 or below −1, and if they did not meet these same thresholds in the *nanos*-GAL4 data.

#### Gene ontology

Gene ontology analysis was carried out in Gene Ontology enRIchment anaLysis and visuaLizAtion tool (GOrilla)^41,42^. The running mode used two unranked lists of genes: (1) enriched transcript list converted to their gene IDs, (2) transcripts that were not filtered out by edgeR’s filterByExprfunction converted to their gene IDs. *D. melanogaster* genes under the GO term ‘signal transduction’ (GO:0007165) were accessed via FlyBase’s controlled vocabulary tool.

#### Motif enrichment

Discriminative regular expression motif elicitation (DREME) from MEME-Suite (MEME version 5.1.0, with Python version 2.7.15) was used to carry out the motif enrichment analysis on 5’ and 3’-UTRs^43^. Control sequences were generated from the list of transcripts that were not filtered out by edgeR’s filterByExpr function. DREME was implemented using the -norc flag, so only the strand given was searched and not the complementary sequences, -rna flag was used to indicate the sequences were of RNA not DNA, and -m was set to 25 to stop searching after 25 motifs had been found.

#### Principal component analysis

Principal component analysis (PCA) was performed using the PCAtools (version 1.2.0)^44^ package in R. PCA was carried out on the triplicate HOW(S)- HA pull-down samples with the log_2_(counts per million) from the transcript-level data, as calculated by the edgeR package in section 2.6.3.4. The pca function was used with the removeVar parameter set to 0.1, which removes the lower 10% of variables based on variance. The biplot function was used to generate a graph of PC1 against PC2.

#### Read summarisation

To summarise the genomic features, the filtered reads were aligned to the *D. melanogaster* genome release 6.22 using Subread. The type parameter (-t) was set to 0 to indicate RNA-seq reads, and the remaining parameters were left at their default settings. featureCounts was used to count reads to the following features: ‘gene’, ‘5UTR’, ‘CDS’ and ‘3UTR’^45^. The GTF file used for this was from *D. melanogaster* genome release 6.22, parameter -s was set to 1 to indicate the library preparation results in a stranded library, -g was used to group features into the gene ID meta-feature. The remaining parameters were left at their default settings. Count tables from the ‘gene’ feature were used in Supplementary Data 1.

### Protein expression and purification

Residues 72–266 of *D. melanogaster* HOW (HOW-PB; FBpp0083576), the STAR domain, was codon optimised and ordered from Genewiz and cloned into the pOPIN-J vector^46^. The His_6_-GST-STAR domain was expressed in BL21(DE3) cells overnight at 18 °C. The fusion protein was affinity-purified with a HisTrap column (GE Healthcare), followed by cleavage with 3C protease overnight. The cleavage products were applied to a HisTrap column to remove the His_6_-GST tag. The cleaved STAR domain was further purified by size exclusion chromatography (SEC) using a 26/600 Superdex 75 column and SEC buffer (Supplementary Table 1).

### Fluorescence anisotropy

RNA oligonucleotides were synthesised by Integrated DNA Technologies. Oligos were 3’ labelled with 6-carboxyfluorescein, and all oligos were desalted after synthesis. Binding assays between fluorescein-labelled oligos and purified STAR protein were carried out in triplicate in black 384-well OptiPlates (Perkin Elmer), and control wells (with no RNA) were carried out once. Approximately 20 μL of RNA binding buffer (Sup Table 3) was added to every well. Approximately 20 μL of 4 nM protein was added to the first column and titrated across each row. Finally, 20 μL of 5 nM fluorescein-labelled RNA oligo was added to the appropriate rows, and for the control rows, 20 μL of RNA binding buffer was added instead. The plate was left to equilibrate for at least 45 min. Then, data were collected on a Spark 10 M Multimode Microplate Reader (Tecan) with a 485 nm (20 nm bandwidth) excitation filter, and parallel (S) and perpendicular (P) channel emission filters at 535 nm (25 nm bandwidth).

Anisotropy values were calculated using the emission values for S and P signals (corrected with the control values from protein-only wells) with the following equation:

$${Anistropy}=\frac{S-P}{S+2P}$$

The logistic function in OriginPro 2020 V2 was first used to determine the theoretical minimum and maximum anisotropy values (A_1_ and A_2_, respectively), using the equation below:

$$y={A}_{2}+\frac{{A}_{1}-{A}_{2}}{1+{\left(\frac{x}{{x}_{0}}\right)}^{p}}$$

Where $y$ is either anisotropy or fraction bound, $x$ is protein concentration, ${x}_{0}$ is the dissociation constant and $p$ is the Hill coefficient. A_1_ and A_2_ were used to calculate the fraction of RNA bound. The logistic function was used again to fit a curve for the fraction bound data. The dissociation constant (x_0_) from these curves was reported as the apparent *K*_D_.

### Cell culture

*D. melanogaster* S2 cells were maintained as previously described^47^. Briefly, S2 cells were grown in Schneider’s medium containing L-glutamine (Lonza), supplemented with 10% (v/v) FBS and 1% (v/v) Penicillin/Streptomycin (GE Healthcare). Cells were maintained at 26 °C in non-vented adherent flasks (Sarstedt).

For RNAi experiments, 2 × 10^6^ cells were seeded per well of a six-well plate. Cells were allowed to settle for 1 h before dsRNA was added. Cells were incubated with dsRNA for 3 days before they were harvested for downstream analysis. Cells were harvested by washing the well with Schneider’s medium. Each cell suspension was split into two, and the cells were pelleted. After removing the medium, RNA and protein were extracted from the two cell pellets.

### RNA extraction and cDNA preparation

RNA was extracted using the Quick-RNA miniprep kit (Zymo). Contaminating gDNA was digested using 24 U/mL Turbo DNAse (Invitrogen), and the RNA was cleaned up using an RNA clean and concentrator kit (Zymo). cDNA was generated using the qScript cDNA synthesis kit (Quantabio).

### PCR, purification of PCR products and qPCR

PCR was performed using recombinant Taq Polymerase (Thermo Fisher, EP0402) as per the manufacturer’s instructions in a T100 Thermal Cycler (BioRad). When performing PCR reactions to amplify RNAi cassettes, where primers contained a T7 promoter and an isoform-specific segment (Supplementary Table 4), the PCR programme consisted of ten cycles at 54 °C and 25 cycles at 65 °C. PCR products were gel extracted using QIAquick gel extraction kit (Qiagen), and large PCR master mixes were concentrated using DNA Clean and Concentrator kit (Zymo).

qPCR was performed using PowerUp SYBR Green Master Mix (Thermo Fisher) in a CFX Connect Thermal Cycler (BioRad). Primers were designed to amplify exon-exon junctions where possible (Supplementary Table 4). mRNA levels were assessed using the DDCq method.

### In vitro transcription

dsRNA was transcribed from PCR products that contained T7 promoters using the TranscriptAid T7 High Yield Transcription kit (Thermo Fisher). Reactions were performed at 37 °C for 3 h. Template DNA was removed, and the RNA was concentrated as above. Annealing buffer was added to the dsRNA (final conc. 10 mM Tris-HCl pH 7.5, 100 mM NaCl) before being heated to 95 °C for 5 min and then cooled at room temperature for 15 min. A total of 10 mg dsRNA was incubated with S2 cells for 3 days.

### Protein extraction and quantification

Pelleted cells were resuspended in RIPA buffer (Abcam) containing 125 U of Benzonase (Sigma-Aldrich). Lysates were incubated on ice for 30 min with occasional agitation before being centrifuged at 17,000×*g* for 5 min at 4 °C. Supernatant was transferred to a fresh tube, and protein concentration was quantified via Pierce Bicinchoninic acid assay (Thermo Fisher). Absorbance was measured at 560 nm in a GloMax Discover Microplate Reader (Promega).

### Mass spectrometry

#### S-TRAP^TM^ digestion

Complex cell lysates suspended in sample buffer and then subjected to S-TRAP^TM^ digestion, as per the instructions (PROTIFI, NY, USA). Briefly, Samples were initially reduced and alkylated using 20 mM DTT for 10 min and 40 mM IAA for 30 min, respectively, followed by acidification of samples using 5.5% phosphoric acid. Acidified samples were then trapped on S-TRAP columns after the addition of sample buffer (100 mM TEAB in 90% methanol) and 1 µg trypsin. Followed by washing with sample buffer, S-TRAP is covered with trypsin and incubated at 47 ˚C for one and half hour. Eluted peptides were concentrated in the SpeedVac concentrator and reconstituted in 0.1% FA.

#### LC-MS analysis of peptides

LC-MS/MS analyses of peptide mixtures were done using a Vanquish Neo UHPLC system connected to an Orbitrap Eclipse Tribrid mass spectrometer (Thermo Fisher Scientific). Prior to LC separation, tryptic digests were online concentrated and desalted using a trapping column (300 μm × 5 mm, μPrecolumn, 5-μm particles, Acclaim PepMap100 C18, Thermo Fisher Scientific) at room temperature. After washing of trapping column with 0.1% formic acid (FA), the peptides were eluted (flow rate – 0.25 nl/min) from the trapping column onto an analytical column (EASY spray column, Acclaim Pepmap100 C18, 2-µm particles, 75 μm × 500 mm, Thermo Fisher Scientific) at 45 °C by a 120 min linear gradient programme (2–50% of mobile phase B; mobile phase A: 0.1% FA in water; mobile phase B: 0.1% FA in 80% ACN). Equilibration of the trapping column and the analytical column was done prior to sample injection into the sample loop. The analytical column with the emitter was directly connected to the ion source.

MS data were acquired in a data-independent strategy (DIA). The survey scan range was set to *m/z* 400–1000 with a resolution of 60,000 (at *m/z* 200) with a standard target value and maximum injection time of 100 ms. HCD MS/MS (30% normalised fragmentation energy) spectra were acquired for a maximum injection time of 55 ms and resolution of 15,000 (at *m/z* 200). The isolation window was set to 15 *m/z*.

#### Mass spectrometry data analysis

The mass spectrometric RAW data files were analyzed using DIA-NN 1.8.1 software. Spectral library was initially generated using cRAP database containing protein contaminants like keratin, trypsin, etc., and UniProtKB protein database for Drosophila melanogaster (taxon: 7227; downloaded 05.2024, version 2024/05, number of protein sequences: 3738). Oxidation of methionine, deamidation (N, Q) and acetylation (protein N-terminus) as optional modifications and carbamidomethyl on cysteine as static modification were used. Trypsin (full) enzyme with two allowed miss cleavages were set. Mass spectra were searched using precursor ion tolerance 10 ppm and fragment ion tolerance 0.02 Da. Peptides and proteins FDR threshold set to <0.01, and proteins with at least one peptide were taken for further analysis.

Output from the DIA-NN software was further processed using the KNIME software container environment (https://github.com/OmicsWorkflows), version 4.7.7a. The processing workflow is available upon request. Briefly, it covered: (a) removal of decoy hits and contaminant protein groups, (b) protein group intensities log2 transformation, (c) LoessF normalisation and (d) differential expression using LIMMA statistical test.

All analysis were performed with R v4.4.0. Before any filtering—cheques were done to check for RNAi off-target effects—the RNAi sequence, including reversing the sequence, complement of the sequence and reverse complement of the sequence was obtained. All possible transcripts associated with the proteins identified in mass-spec, before any filtering, were obtained (no pADJ and no LFC filtering performed). Pairwise alignments were performed between the resulting transcripts and the RNAi sequence(s) to look for any off-target effects, while allowing for incomplete alignment (local alignments, global alignments and short indels allowed). No successful alignments were determined. Also checked the RNAi sequence against the full transcript DB, to ensure nothing was missed.

### Identification of motifs in mass spectrometry hits

The resulting mass-spec data were filtered with a *p*^ADJ^ < 0.05 and an absolute log_2_ fold change of >1. The remaining identified proteins of interest were mapped to their appropriate transcript IDs with the BioConductor R package AnnotationDbi^48^ and the genome annotation for the fly database. In the case of a protein mapping to multiple transcript IDs, the transcript consisting of the longest 5’ UTR was retained. Both the 5’ UTR and 3’ UTR sequences for all identified transcripts were obtained using the genome fly annotation. DREME from the meme-suite was used on the obtained UTRs with the following flags: -norc, -rna, -m 25^49,50^.

### Analysis of previously identified motifs

The previously filtered and transcript ID-mapped protein hits from Mass-Spec were utilised to search for previously determined enriched motifs (5’ UTR - GCG(A/U)G and 3’ UTR - (A/U/G)CUAAC) were searched for in the obtained UTR sequences. The mass spectrometry protein hits were also mapped to their gene IDs, all 5’ UTR and 3’ UTR sequences obtained, and a subsequent search was undertaken with the enriched 5’ UTR and 3’ UTR gene motifs, GCG(A/G/C)A and UACU(A/C)(A/G), respectively.

### Overlap with less stringent RIP-Seq results

Three forms of analysis were performed to identify a common overlap. The Mass-Spec data was filtered as follows: (i) no filtering/stringency of any kind, (ii) filtering by *p*^ADJ^ < 0.05 only and (iii) filtering by *p*^ADJ^ < 0.05 and an absolute log_2_ fold change >1. Proteins identified in the filtered and unfiltered Mass-Spec data were mapped to fly annotation gene IDs using the BioConductor R package AnnotationDbi^48,50^ and the genome annotation for the fly database. Where transcript IDs were the primary identifier for the RIP-Seq results, these were mapped to their gene IDs using the same methods. A search for the gene IDs obtained from the three different, previously described mass-spec filtering conditions was performed using the gene IDs from the RIP-Seq results.

### Reporting summary

Further information on research design is available in the Nature Portfolio Reporting Summary linked to this article.

## Supplementary information

Supplementary Information

Description of Additional Supplementary Files

Supplementary Data 1

Supplementary Data 2

Reporting Summary

## Data Availability

RIP-seq data have been deposited to the Gene Expression Omnibus with accession ID: GSE201319. Summary of the 121 HOW(S) bound mRNAs, their enrichment level, expression level, motifs contained and association with signal transduction GO terms can be found in Supplementary Data 1. Mass spectrometry data have been deposited in PRIDE with accession PXD070902. Summary of the mass-spec data relating to the HOW(S) targets can be found in Supplementary Data 2.

## Code availability

Any code in addition to the tools included in the supplementary methods is available at https://github.com/magapiou/HOW_RIPseq.
