# Supplementary material for: Held out wings RNA binding activity in the cytoplasm during early spermatogenesis
Source: Commun Biol. 2026 Jan 12;9:156. doi: 10.1038/s42003-025-09435-4 (PMC12868882; doi:10.1038/s42003-025-09435-4)
Supplement: Supplementary file 1 — Supplementary Information [file 42003_2025_9435_MOESM1_ESM.pdf]

Supplementary Figure 1

A

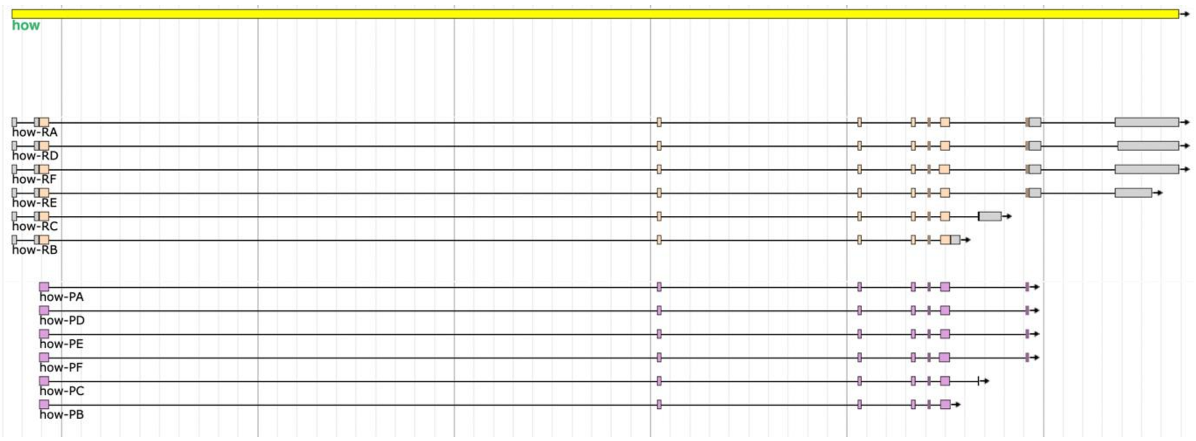

B

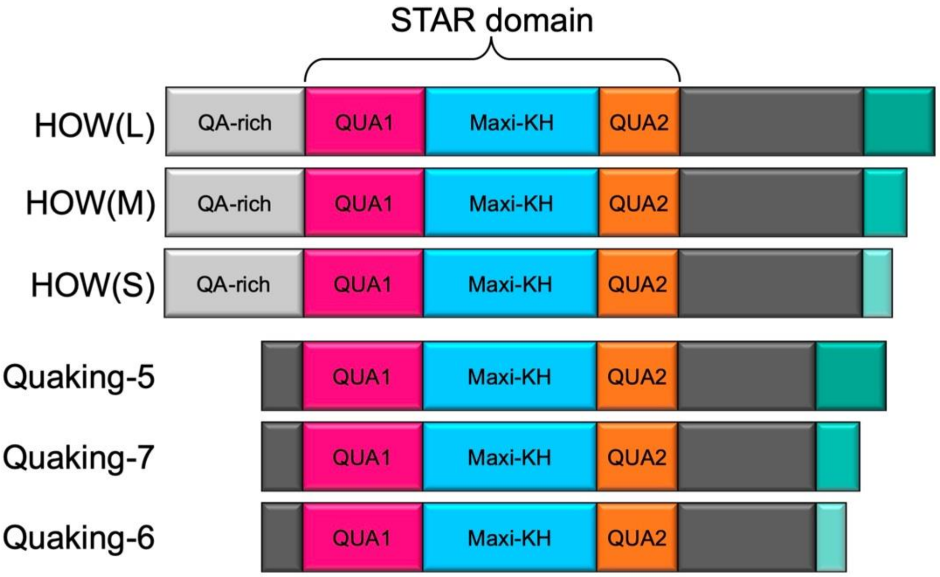

Supplementary Figure 1: Isoforms of HOW and its human ortholog quaking
(A) Genomic structure of HOW and its RNA and protein isoforms in FlyBase. HOW(S) corresponds to how-PB translated from how-RB. HOW(L) corresponds to how-PA, PD, PE and PF. (B) Schematic showing different protein isoforms of HOW (S, M, L) and its human ortholog Quaking (5, 6 and 7). Quaking-6 is orthologous to HOW(S) and its also cytoplasmic.

Supplementary Figure 2

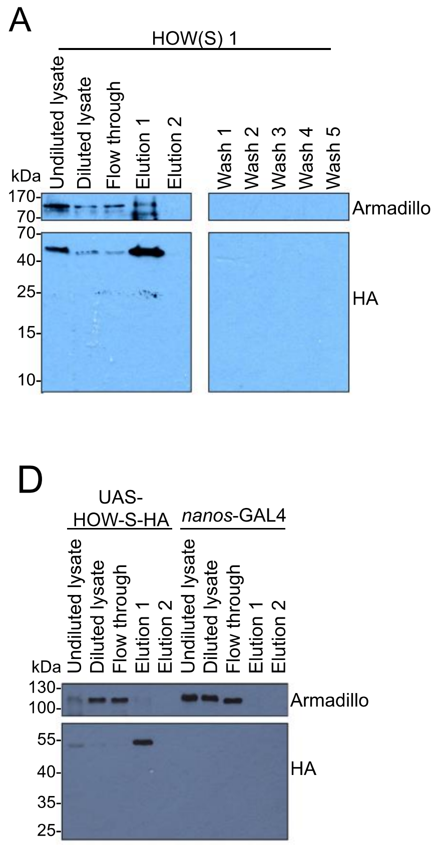

Supplementary Figure 2: Expression and pull-down of HA-tagged HOW(S) from cytoplasm of germ cells

Western blots of HOW(S)-HA pull-down from both experimental *nanos*-GAL4>HOW(S)-HA flies (A-C) and parental control lines (D). Elution 1 and 2 refer to the two sequential rounds of elution performed. Lack of signal in elution 2 indicates that all bound protein was eluted in elution 1.

Supplementary Figure 3

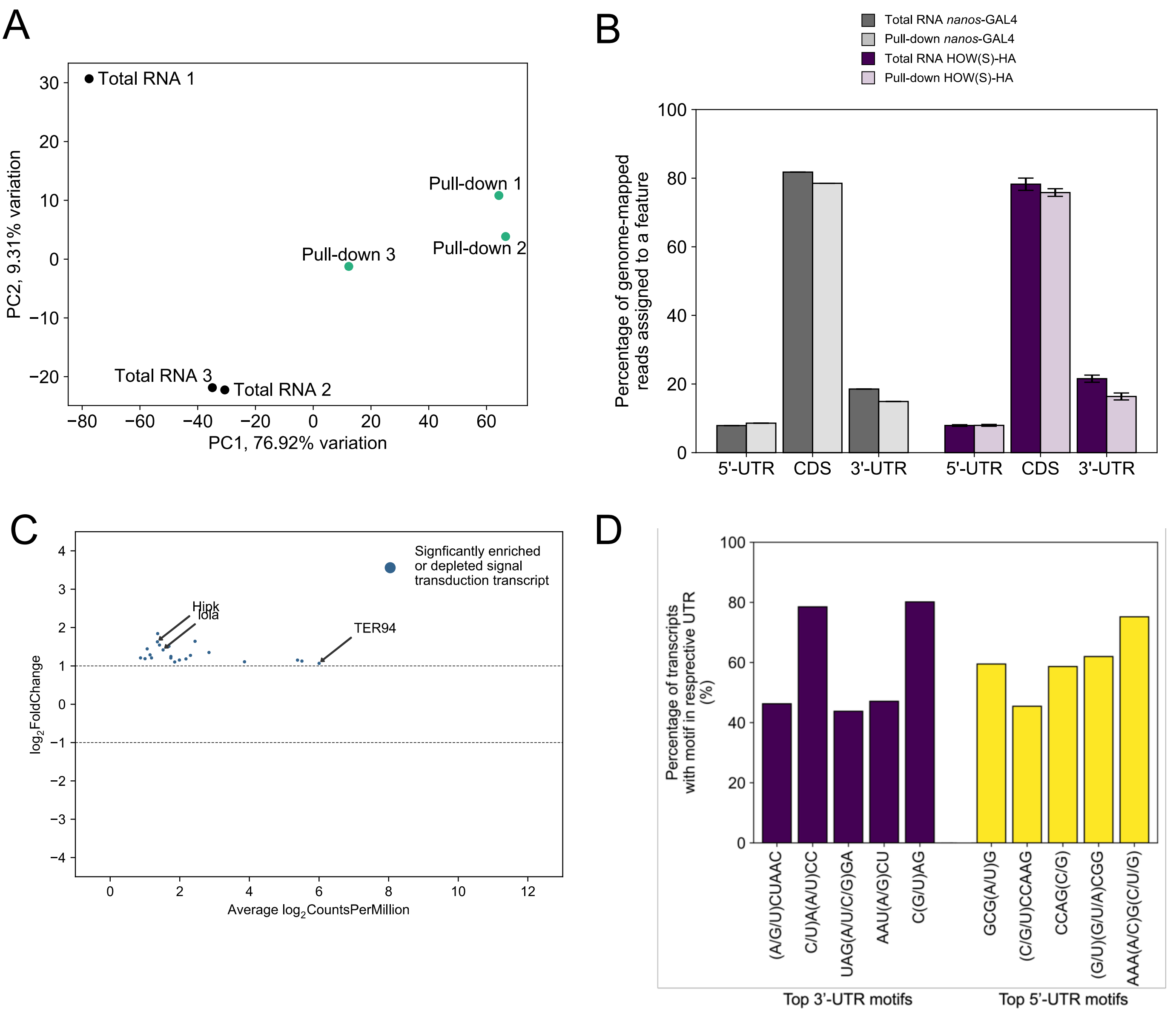

Supplementary Figure 3: Enrichment of HOW(S) bound RNA, with role in signal transduction

A) Biplot from PCA using the $\log_2$(Counts Per Million) of the transcriptome quasi-mapped reads. B) Reads from HOW(S) and parental *nanos*-GAL4 RIP-Seq map across mRNA transcripts (5’-UTRs, CDSs and 3’-UTRs) at similar levels in the input and pull-down samples (error bars are SEM). C) Enriched mRNAs identified at transcript level whose protein products function in signal transduction marked in blue and 3 specific mRNAs of interest labelled. D) Percentage of RIP-Seq bound transcripts containing the top motifs in either their 3’-UTR or 5’-UTR.

Supplementary Figure 4

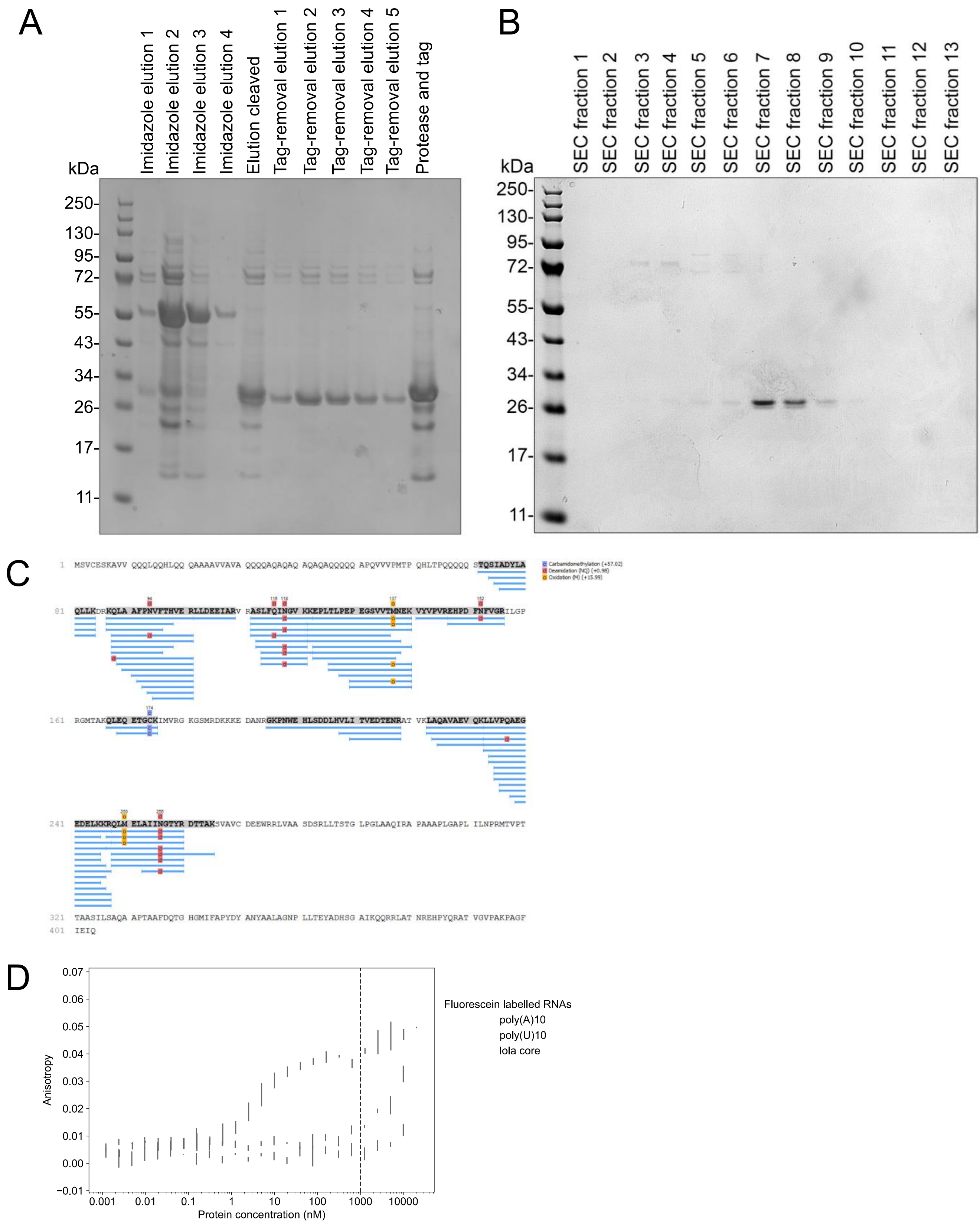

Supplementary Figure 4: HOW KH domain binds to HOW(S) targets with high affinity and specificity

A-B) Coomassie stained gels from the purification of HOW-STAR domain. A) Ni^{2+} affinity chromatography to purify His_6-GST tagged HOW-STAR. Imidazole elutions were combined and cleaved overnight ('elution cleaved') and the tag and protease were removed by another round of Ni^{2+} affinity chromatography. B) Further purification of HOW-STAR performed by size exclusion chromatography. C) Mass spectrometry of purified HOW-STAR shows coverage across residues 72-265 (STAR domain). D) FA of HOW-STAR with 3 RNA oligos. Above 1000 nM of protein suspected non-specific binding occurs.

Supplementary Figure 5

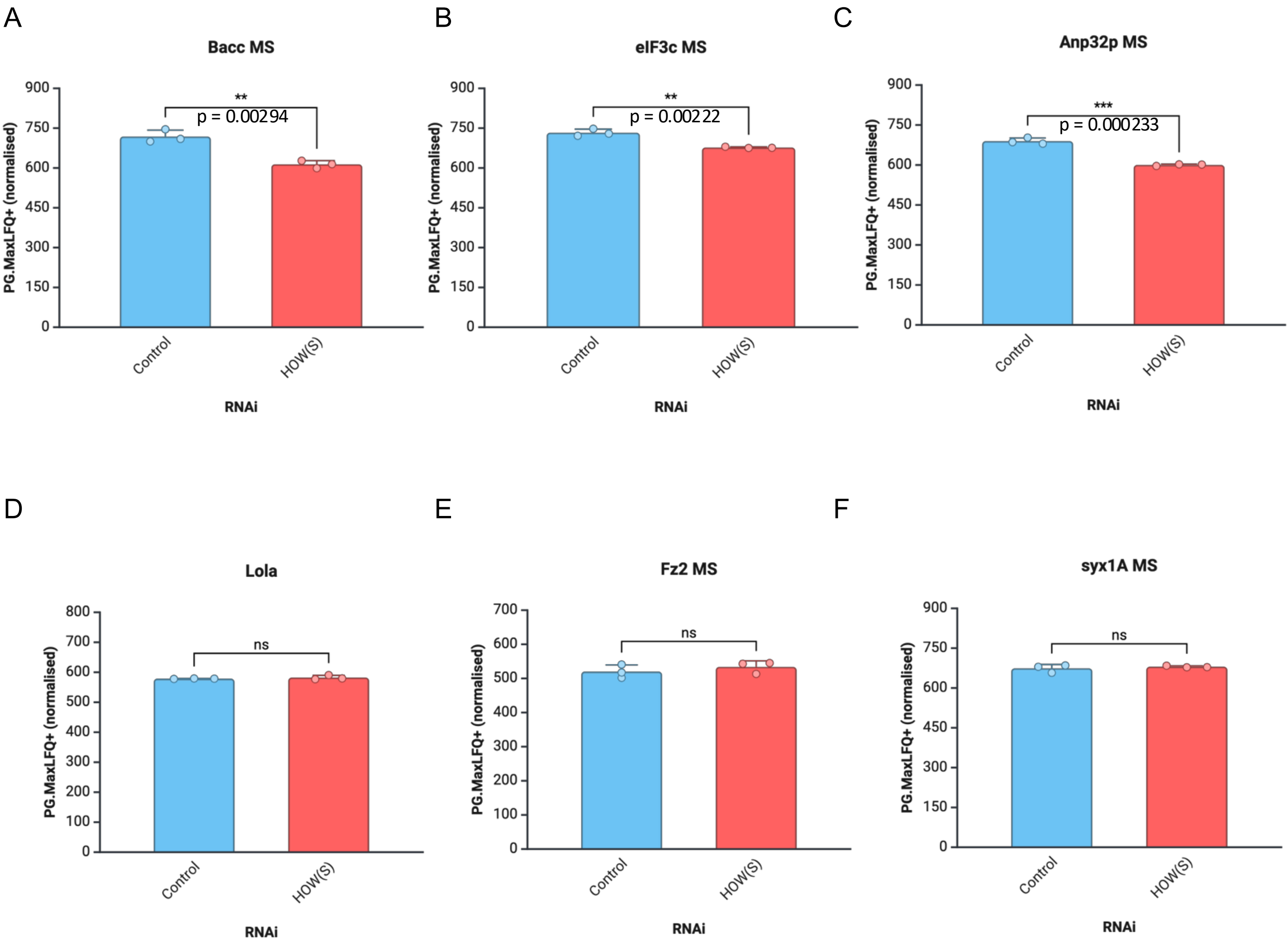

Supplementary Figure 5: Impact of HOW(S) RNAi knockdown in S2 cells on protein levels
Plots of protein quantification (Normalised PG.MaxLFQ+) from mass spectrometry for in Control and HOW(S) RNAi treated cells. (A-C) are from mass spectrometry in S2 cells, which contained HOW motifs in their corresponding mRNA transcripts and had RT-qPCR also performed; (A) Anp32p, (B) Bacc, (C) eIF3c. (D-F) are targets identified from testis RIP-Seq; (D) Lola, (E) Fz2, (F) synx1A. T-tests were performed and p values **<0.003, and ***<0.001 calculated and reported.

Supplementary Table 1 – Buffers and solutions used. All descriptions are for 1X solutions unless stated otherwise.

Buffer name	Buffer components
PBX	0.1% (v/v) Triton X-100, 0.5% (v/v) normal goat serum (Thermo Fisher), in 1X phosphate buffered saline (PBS)
Blocking buffer	0.1% Triton X-100, 2% (v/v) normal goat serum, in 1X PBS
NT2 buffer	50 mM Tris-HCl pH 7.4, 150 mM NaCl, 1 mM MgCl2, 0.05% (v/v) IGEPAL
PBS-T	0.1% Tween 20, in 1X PBS
2X protein sample buffer	125 mM Tris-HCl pH 6.8, 5% (w/v) SDS, 25% (v/v) glycerol, 10% (v/v) β-mercaptoethanol, 0.004% (w/v) bromophenol blue
RIP lysis buffer	50 mM Tris-HCl pH 8, 150 mM NaCl, 10 mM MgCl2, cOmplete mini protease inhibitor cocktail (Roche), 1% (v/v) IGEPAL, 24 U/mL Turbo DNase (Thermo Fisher), 30 U/mL RNasin Plus RNase inhibitor (Promega)
SEC buffer	25 mM HEPES pH 7.6, 150 mM NaCl and 1 mM TCEP
RNA binding buffer	20 mM Tris-HCl pH 7.5, 150 mM NaCl, 0.01% Triton X-100

Supplementary Table 2 – Antibodies used for immunofluorescence. Primary antibodies are listed in the top half of the table and secondary antibodies in the bottom half.

**Antibody**	**Additional information**	**Stock concentration (µg/mL)**	**Dilution used**	**Supplier (catalogue reference)**
Vasa	Rat IgM<br>Monoclonal<br>Hybridoma supernatant	44	1:200	DSHB<br>(anti-vasa)
HA	Mouse IgG2b<br>Monoclonal<br>Ascites fluid	400	1:100	Roche<br>(12CA5)
Goat anti-rat IgM	Alexa Fluor 488<br>Polyclonal<br>Affinity purified	2000	1:400	Thermo Fisher<br>(A-21212)
Goat anti-mouse IgG2b	Alexa Fluor 594<br>Polyclonal<br>Affinity purified	2000	1:400	Thermo Fisher<br>(A-21145)

Supplementary Table 3 – Antibodies used for western blotting. Primary antibodies are listed in the top half of the table and secondary antibodies in the bottom half.

Antibody	Additional information	Stock concentration (µg/mL)	Dilution used	Supplier (catalogue reference)
Armadillo	Mouse IgG2a Monoclonal Hybridoma supernatant	27	1:1000	DSHB (N2 7A1)
HA	Rabbit IgG Polyclonal Affinity purified	1000	1:5000	Abcam (ab9110)
Horse anti-mouse IgG	HRP-linked	153	1:5000	Cell Signalling Technology (7076S)
Goat anti-rabbit IgG	HRP-linked	65.7	1:5000	Cell Signalling Technology (7074S)

Supplementary Table 4 – PCR Primers. Used for RNAi duplex PCR, PCR and qPCR. Target names as referred to in text along with sequences of forward and reverse primers.

Primer Set	Forward primer	Reverse primer
BSK RNAi	TAATACGACTCACTATAGGGAGGAGTT AAAATTCGCGTTAAATTT	TAATACGACTCACTATAGGGAGGA GTGTGGTGGTTACGCGCAGCG

HOW(S) RNAi	TAATACGACTCACTATAGGGAGGGTG GGTTGTTTGCCAGATAATC	TAATACGACTCACTATAGGGAGG GGATGAATGATTCGCTTAGTG
HOW(S) PCR	TGGGTTGTTTGCCAGATAATCACAT	GGAAAAACAAACGTACCGGGC
HOW(L) PCR	GGGGCGAAACAGAAACAACA	TGCGTTGATTGCTTTGTACCA
HOW All PCR	TCTCTTTCTTTGCGCACGGT	CTTCTTGTCGCGCATGGAAC
GAPDH PCR	TCGACTCACGGTCGTTTCAA	CCAAGCGGACGGTAAGATCC
Fz2 qPCR	TTCGGGATTAACGGCGGAAA	CAAACGGCGTGGTCTAACATTT
Rhea qPCR	AAATCTGACGGAGCCACAGC	AGGGTGTTCTCACGCCATTC
Lola qPCR	CAACGGGATCCCCAAGACTTC	TTGATCACCGGCGTTCCAAT
Syx1A qPCR	AGAATTCGGAAGACGCAGCA	ACGCTGTATCCTTCCCTTGC
Anp32a qPCR	CGCACGCAAAGTAAATCAGATCA	AAGCGATTCCAAGGCTGTGT
Bacc qPCR	CAAGAGGCCAGCAGAAGCCA	GGGGATGTCGTACTCGTCGC
eIF3c qPCR	TGTCGCGTTTCTTTGCCAAC	TCGTCGCTGAATTGGAAGGC
GAPDH qPCR	TGAACGGCCAGAAGATCACC	CTCCACCACATACTCGGCTC
U1 qPCR	CTCCGGAGTGAGGCTTGG	ACGCACGAGTTATTCACATTAGG
