# Supplementary material for: Held out wings RNA binding activity in the cytoplasm during early spermatogenesis
Source: Commun Biol. 2026 Jan 12;9:156. doi: 10.1038/s42003-025-09435-4 (PMC12868882; doi:10.1038/s42003-025-09435-4)
Supplement: Supplementary file 2 — Description of Additional Supplementary Files [file 42003_2025_9435_MOESM2_ESM.docx]

**Description of Additional Supplementary Files**

File name: Supplementary Data 1
Description: Results of RIP-Seq analysis

File name: Supplementary Data 2
Description: Motif analysis on mass spec hits
