# Supplementary material for: Held out wings RNA binding activity in the cytoplasm during early spermatogenesis
Source: Commun Biol. 2026 Jan 12;9:156. doi: 10.1038/s42003-025-09435-4 (PMC12868882; doi:10.1038/s42003-025-09435-4)
Supplement: Supplementary file 5 — Reporting Summary [file 42003_2025_9435_MOESM5_ESM.pdf]

nature portfolio

Corresponding author(s): Julie L Aspden

Last updated by author(s): 14/8/25

Reporting Summary

Nature Portfolio wishes to improve the reproducibility of the work that we publish. This form provides structure for consistency and transparency in reporting. For further information on Nature Portfolio policies, see our Editorial Policies and the Editorial Policy Checklist.

Statistics

For all statistical analyses, confirm that the following items are present in the figure legend, table legend, main text, or Methods section.

n/a	Confirmed	
☐	☒	The exact sample size (*n*) for each experimental group/condition, given as a discrete number and unit of measurement
☐	☒	A statement on whether measurements were taken from distinct samples or whether the same sample was measured repeatedly
☐	☒	The statistical test(s) used AND whether they are one- or two-sided<br>*Only common tests should be described solely by name; describe more complex techniques in the Methods section.*
☒	☐	A description of all covariates tested
☒	☐	A description of any assumptions or corrections, such as tests of normality and adjustment for multiple comparisons
☐	☒	A full description of the statistical parameters including central tendency (e.g. means) or other basic estimates (e.g. regression coefficient) AND variation (e.g. standard deviation) or associated estimates of uncertainty (e.g. confidence intervals)
☐	☒	For null hypothesis testing, the test statistic (e.g. *F*, *t*, *r*) with confidence intervals, effect sizes, degrees of freedom and *P* value noted<br>*Give P values as exact values whenever suitable.*
☒	☐	For Bayesian analysis, information on the choice of priors and Markov chain Monte Carlo settings
☒	☐	For hierarchical and complex designs, identification of the appropriate level for tests and full reporting of outcomes
☒	☐	Estimates of effect sizes (e.g. Cohen's *d*, Pearson's *r*), indicating how they were calculated

Our web collection on statistics for biologists contains articles on many of the points above.

Software and code

Policy information about availability of computer code

Data collection	NA
Data analysis	Cutadapt (version 1.1), Galaxy (version 1.0.2) , Subread (version 2.0.0) , Salmon (version 0.14.2) , RStudio, version 0.99.486, with R version 3.6.2 , tximport package (version 1.14.0), edgeR package (version 3.28.0), Gene Ontology enRIchment anaLysis and visuaLizAtion tool , Discriminative Regular Expression Motif Elicitation (DREME) from MEME Suite (MEME version 5.1.0, with Python version 2.7.15) , PCAtools (version 1.2.0), ucsc-gtftogenepred package (version 366), Spliced Transcripts Alignment to a Reference (STAR; version 2.7.3a) , OriginPro 2020 V2, KNIME software container environment (https://github.com/OmicsWorkflows), version 4.7.7a, BioConductor R package AnnotationDbi.<br><br>Custom code for simple tasks is available on request

For manuscripts utilizing custom algorithms or software that are central to the research but not yet described in published literature, software must be made available to editors and reviewers. We strongly encourage code deposition in a community repository (e.g. GitHub). See the Nature Portfolio guidelines for submitting code & software for further information.

Data

Policy information about availability of data

All manuscripts must include a data availability statement. This statement should provide the following information, where applicable:

- Accession codes, unique identifiers, or web links for publicly available datasets
- A description of any restrictions on data availability
- For clinical datasets or third party data, please ensure that the statement adheres to our policy

RIP-seq data have been deposited to the Gene Expression Omnibus with accession ID: GSE201319. Summary of the 121 HOW(S) bound mRNAs, their enrichment level, expression level, motifs contained and association with signal transduction GO terms, can be found in Sup data file.

Human research participants

Policy information about studies involving human research participants and Sex and Gender in Research.

Reporting on sex and gender	NA
Population characteristics	NA
Recruitment	NA
Ethics oversight	NA

Note that full information on the approval of the study protocol must also be provided in the manuscript.

Field-specific reporting

Please select the one below that is the best fit for your research. If you are not sure, read the appropriate sections before making your selection.

☒ Life sciences ☐ Behavioural & social sciences ☐ Ecological, evolutionary & environmental sciences

For a reference copy of the document with all sections, see nature.com/documents/nr-reporting-summary-flat.pdf

Life sciences study design

All studies must disclose on these points even when the disclosure is negative.

Sample size	Triplicate for RIP-Seq and FA
Data exclusions	No data excluded
Replication	Conclusions based on triplicates for RIP-Seq with each samples containing 1000 pairs of testes
Randomization	NA
Blinding	NA

Reporting for specific materials, systems and methods

We require information from authors about some types of materials, experimental systems and methods used in many studies. Here, indicate whether each material, system or method listed is relevant to your study. If you are not sure if a list item applies to your research, read the appropriate section before selecting a response.

Materials & experimental systems

n/a	Involved in the study
☐	☒ Antibodies
☒	☐ Eukaryotic cell lines
☒	☐ Palaeontology and archaeology
☐	☒ Animals and other organisms
☒	☐ Clinical data
☒	☐ Dual use research of concern

Methods

n/a	Involved in the study
☒	☐ ChIP-seq
☒	☐ Flow cytometry
☒	☐ MRI-based neuroimaging

Antibodies

Antibodies used

Armadillo Mouse IgG2a (Monoclonal); Hybridoma supernatant (1:1000) DSHB(N2 7A1)
HA Rabbit IgG (Polyclonal); Affinity purified (1:5000) Abcam (ab9110)
Horse anti-mouse IgG HRP-linked (1:5000) Cell Signalling Technology (7076S)
Goat anti-rabbit IgG HRP-linked (1:5000) Cell Signalling Technology (7074S)
Vasa Rat IgM; MonoclonalHybridoma supernatant (1:200) DSHB (anti-vasa)
HA Mouse IgG2b ;MonoclonalAscites fluid (1:100) Roche (12CA5)
Goat anti-rat IgM Alexa Fluor 488; Polyclonal Affinity purified (1:400) Thermo Fisher (A-21212)
Goat anti-mouse IgG2b Alexa Fluor 594 Polyclonal; Affinity purified (1:400) Thermo Fisher (A-21145)

Validation

Spatial expression of the Drosophila segment polarity gene armadillo is posttranscriptionally regulated by wingless. Wieschaus ECell 63.3 (1990 Nov 2): 549-60.

Vasa: Reduced fertility of Drosophila melanogaster hybrid male rescue (Hmr) mutant females is partially complemented by Hmr orthologs from sibling species.Barbash DA Genetics 181.4 (2009 Apr): 1437-50.

Animals and other research organisms

Policy information about studies involving animals; ARRIVE guidelines recommended for reporting animal research, and Sex and Gender in Research

Laboratory animals	UAS-HOW-S-HA line generously gifted by Prof T. Volk, nanos-GAL4 line used is #64277 from BDSC.
Wild animals	NA
Reporting on sex	Males-since study is focused on role in testes
Field-collected samples	NA
Ethics oversight	NA

Note that full information on the approval of the study protocol must also be provided in the manuscript.
